# Supplementary material for: Imaging plant germline differentiation within Arabidopsis flowers by light sheet microscopy
Source: eLife. 2020 Feb 11;9:e52546. doi: 10.7554/eLife.52546 (PMC7012603; doi:10.7554/eLife.52546)
Supplement: Supplementary file 1. — (A) Oligonucleotides used in the study. (B) Overview of image processing. [file elife-52546-supp1.docx]

***Supplementary Material***

**Supplementary Table 1.** List of oligonucleotides

| **Primer** | **Sequence [5’-3’]** | **Application** |
| --- | --- | --- |
| **AtPCNA1_f** | CACCGGGCAAAGTCGGTTTTGGA | Cloning PCNA1 construct |
| **AtPCNA1_r** | GGGATTAGTGTCTTCTTCTTCTTCA | Cloning PCNA1 construct |
| **P1_ASY1** | TACTATTTATCTGGCCATGGCGGCCTTCGACTTGTAGACTAGAGAG | Cloning ASY1 construct |
| **P2_ASY1** | ACCTCCACCTCCAGCTCCAGGCCGGCCGGAGTACTGCAGAATAGGCTC | Cloning ASY1 construct |
| **P3_ASY1** | GCGGCCGCTGCCGGGGCTGGGCGCGCCAAGCGTCAGAAATCTCAAGC | Cloning ASY1 construct |
| **P4_ASY1** | ATTAGTCGCAATGGCCCTTAAGGCCAAGCCCAATAACGTACATGC | Cloning ASY1 construct |
| **P1_H2B3G** | TACTATTTATCTGGCCATGGCGGCCAGCTACTGTGATGGCTCCTACACG | Cloning H2B construct |
| **P2_H2B3G** | ACCTCCACCTCCAGCTCCAGGCCGGCCTACTGGTTTCTCGGCGGCTG | Cloning H2B construct |
| **P3_H2B3G** | GCGGCCGCTGCCGGGGCTGGGCGCGCCGAGGAGAAATCAAAAGCCGAG | Cloning H2B construct |
| **P4_H2B3G** | ATTAGTCGCAATGGCCCTTAAGGCCCTGCCTCTGCTCTCTCTTGTAAAC | Cloning H2B construct |
| **TAGmRuby2_clo_F** | TTAAGGCCGGCCTGGAGCTGGAGGTGGAGGTGGAGCTGGTGTGTCTAAGGGCGAAGAG | Cloning mRuby2 |
| **TAGmRuby2_clo_R** | AATTGGCGCGCCCAGCCCCGGCAGCGGCCGCAGCACCGGCCTTGTACAGCTCGTCC | Cloning mRuby2 |

# **Supplementary Table 2.** Image processing

## **Figures**

|  | **deconvolution** | **Dual side fusion** | **MIP** | **gamma** | **Background subtr** | **Multiview**  **Reconstr.** |
| --- | --- | --- | --- | --- | --- | --- |
| **01 B upper** |  | max | x |  |  |  |
| **01 B lower** | x |  | partial |  | 0.6mm only |  |
| **01 C** | x |  | partial |  |  |  |
| **02 A** |  | max | x |  |  |  |
| **02 B, C** |  | max | x |  |  | X |
| **02 D, E** |  | max | partial |  |  | X |
| **02 F, G** |  | max | x |  |  | X |
| **03 A upper** |  | max | x |  |  |  |
| **03 A lower** |  |  | partial |  |  |  |
| **03 B** |  |  | partial |  |  |  |
| **04 A** |  | max | x | x |  |  |
| **04 B upper** |  | max | x | x |  |  |
| **04 B lower** |  | max | x |  |  |  |
| **05 A** | x |  | partial |  |  |  |
| **05 B** | x |  | partial |  |  |  |
| **05 C** | x |  | partial |  |  |  |
| **05 D** | x |  | partial |  |  |  |
| **05 E** | x |  | partial |  |  |  |
| **06 A** | x |  | partial |  |  |  |
| **06 B** | x |  | partial |  |  |  |
| **07 top** |  |  | partial |  |  |  |
| **07 middle** | x |  | partial |  |  |  |
| **07 bottom** | x |  | partial |  |  |  |
| **S 02 A upper** |  | max | x |  |  |  |
| **S 02 A lower** |  | max | partial |  |  |  |
| **S 02 B upper** |  | max | x |  |  |  |
| **S 02 B lower** |  |  | partial |  |  |  |

## **Videos**

|  | **deconvolution** | **Dual side fusion** | **MIP** | **gamma** | **Drift correction** | **Multiview**  **Reconstr.** |
| --- | --- | --- | --- | --- | --- | --- |
| **01** |  | max | x |  |  | x |
| **02** |  | max | x |  |  |  |
| **03** |  | max | x | x |  |  |
| **04** | x |  | partial |  |  |  |
| **05** | x |  | partial |  |  |  |
| **06** | x |  | partial |  |  |  |
| **07** | x |  | partial |  |  |  |
| **08** | x |  | partial |  |  |  |
| **09** | x |  | partial |  |  |  |
| **10** | x |  | partial |  |  |  |
| **11** |  |  | partial |  |  |  |
| **12** | x |  | partial |  | x |  |
| **13** | x |  | partial |  | x |  |

Dual side fusion – max = maximum fusion

Deconvolution algorithm in methods

MIP = maximum intensity projection: x = all layers, partial = only representable number of layers

Gamma = a non-linear transformation was applied to enhance features for presentation only (ZEN)

Background subtraction = to enhance image background was removed in FIJI

Drift correction = original video contains drift of the sample, removed in FIJI

Display = no bleaching correction was used, only fixed display settings for all frames (in compare to figures – all separately adjusted in brightness/contrast)
